# Seasonal variation influences flavonoid biosynthesis path and content, and antioxidant activity of metabolites in *Tetrastigma hemsleyanum* Diels & Gilg

**DOI:** 10.1371/journal.pone.0265954

**Published:** 2022-04-28

**Authors:** YanShou Shi, Li Yang, MinFen Yu, ZhaoHui Li, ZhiJun Ke, XiaoHua Qian, Xiao Ruan, LiPing He, Feng Wei, YingXian Zhao, Qiang Wang

**Affiliations:** 1 Ningbo Technology University, Ningbo, China; 2 Ningbo Research Institute, Zhejiang University, Ningbo, China; 3 Ningbo Forest Farm, Ningbo, China; 4 Department of Traditional Chinese Medicines, Zhejiang Pharmaceutical College, Ningbo, China; 5 Bureau of Natural Resources and Planning Xianju County, Taizhou, China; Universite d’Orleans, FRANCE

## Abstract

Environmental conditions contribute to plant growth and metabolism. This study aimed to determine a suitable environment and climate for large-scale artificial cultivation of an endangered plant, *Tetrastigma hemsleyanum*, by investigating the seasonal variations influencing the flavonoid biosynthetic selectivity and antioxidant activity of its major metabolites. Under conditions of precipitation (2.0~6.6 mm), temperature (17.5~24.1°C), humidity (67.3~80.2%), and sunshine duration (3.4~5.8 h) from April to May, the total flavonoid content in *T*. *hemsleyanum* reached higher levels between 281.3 and 392.8 μg/g. In the second half of April, the production selectivity (PS) of isoorientin (IsoO), orientin (Or), rutin (Rut), isoquercitin (IsoQ), kaempferol-3-O-rutinoside (Km3rut), astragalin (Ast), quercetin (Qu), apigenin (Ap), and kaempferol (Km) were 0.30, 0.06, 0.07, 0.07, 0.00, 0.04, 0.38, 0.05, and 0.03, respectively. Naringenin was dehydrogenated or hydroxylated to initiate two parallel reaction pathways for flavonoid biosynthesis in *T*. *hemsleyanum*: path I subsequently generated flavone derivatives including apigenin, luteolin, orientin, and isoorientin, and path II subsequently generated flavonol derivatives including Km, Qu, IsoQ, Rut, Ast, and Km3rut. The reaction selectivity of path II (RPS_II_) from January 1 to September 30 was considerably higher than that of path I (RPS_I_), except for March 16–31. In addition, either the content or antioxidant activity of three major metabolites in *T*. *hemsleyanum* followed the order of phenolic compounds > polysaccharides > sterols, and exhibited dynamic correlations with environmental factors. Naringenin favored hydroxylation and derived six flavonol compounds from January to September, and favored dehydrogenation and derived three flavone compounds from October to December. In most months of a year, Km preferentially favored hydroxylation rather than glucosylation.

## 1. Introduction

*Tetrastigma hemsleyanum* Diels & Gilg (*T*. *hemsleyanum*), popularly termed “Sanyeqing,” is a perennial and edible plant of Vitaceae, and is native to China [1, 2]. Geographically, *T*. *hemsleyanum* usually grows under the shade of mountainous cliffs 700 m above sea level and at a favorable temperature of approximately 18°C [2, 3]. The formation of its tubers require 3 to 5 years in the natural environment; the major bioactive constituents in the tuber of *T*. *hemsleyanum* reportedly include various flavonoids, polysaccharides, and terpenes [4–8]. In traditional Chinese herbal medicine, *T*. *hemsleyanum* tuber has been used to eliminate inflammation, improve blood circulation, and even resist viruses and tumors [6, 9–11]. *In vivo* pathological tests of tumor-bearing mice have confirmed the antitumor activity of flavonoids derived from the secondary metabolism of *T*. *hemsleyanum* [5]. Recently, Li *et al*. [12]quantified four main flavonoids that include rutin, quercetin-3-*O*- glucoside (isoquercetin), kaempferol-3-*O*-rutinoside, and kaempferol-3-*O*-glucoside (astragalin) in the tuber of *T*. *hemsleyanum*, and conducted both *in vitro* and *in vivo* tests to demonstrate the potential efficacy of these flavonoids in treating non-small cell lung cancer. In China, however, *T*. *hemsleyanum* is becoming endangered because of the harsh environmental conditions and excessive artificial excavation; hence its propagation has to rely on artificial cultivation in greenhouses by manipulating various environmental factors such as temperature, soil moisture, and illumination intensity.

Environmental conditions significantly affect the biosynthesis of various plant metabolites [13–15]. For most plants, environmental factors such as sunlight, temperature, humidity, precipitation, soil fertility, and salinity can synergistically alter their ability to synthesize metabolites, eventually altering their phytochemical profiles and the production of bioactive substances [16]. For example, light irradiation on *Ipomoea batatas* leaves for 16 h dramatically increased various flavonoids (anthocyanins, catechins, and flavonols) and phenolic acid content (hydroxycinnamic and hydroxybenzoic acids) [17]. In addition, the contents of various polysaccharides, flavones (ginkgetin, amentoflavone, and quercetin), and taxoids (paclitaxel, 10-deacetylbaccatin III, baccatin III, cephalomannine, and 10-deacetyltaxol) in *Taxus wallichiana* var. *mairei* showed seasonal variations [18]. Furthermore, polysaccharide content was the highest at 28.52± 0.57 mg/g in September and the lowest at 9.39± 0.17 mg/g in January, taxoids were the highest at 1.77± 0.38 mg/g in January and the lowest at 0.61± 0.08 mg/g in September, and flavonoid content was the highest in August. Moreover, Helmig *et al*. [19]found that the emission of *β*-caryophyllene, *α*-bergamotene, *α*-farnesene, and β-farnesene from seven pine species increased exponentially with temperature.

The seasonal variations in plant chemical composition and bioactivity are closely related to the different stages of metabolism and changes in climatic factors, such as temperature, soil humidity, and rainfall [18, 20]. Dryness, heat, and light affect flavonoid and phenolic contents in plants [17, 21–23]. High temperatures of 30 to 40°C could inhibit flavonoid biosynthesis by suppressing gene expression and enzyme activity [24], whereas low temperatures usually induce biosynthesis, which may consecutively inhibit flavonoid synthesis in the absence of light [25]. In general, varying climatic environments in both different locations and even in the same location can alter the content of active ingredients in medicinal plants [26]. For example, tanshinones accumulation in plants strongly depends on meteorological factors, such as average relative humidity and annual average temperature [27]. Overall, the correlations between metabolites and environmental factors often display diverse, pluralistic, and dynamic characteristics.

To date, various biotic and abiotic stress factors have been investigated to mainly manipulate the plant metabolite profile [13–16]. Most investigations have been apparently confined to the phenomenological level, because of the limited research methods and the lack of understanding of metabolic mechanisms and micro pathways that are influenced by various factors and activities [28, 29]. Certain plants may produce compounds with adverse effects, and the management of such compounds in medicinal and food plants is attracting increasing attention [30]. Dai *et al*. [2] determined the optimal light intensity for the growth of *T*. *hemsleyanum* by investigating the effect of different shading treatments on chlorophyll content, chlorophyll fluorescence, and photosynthetic capacity. Song *et al*. [31]revealed that endophytic fungi from their calabash-shaped roots could regulate the growth process, expression of expansion genes, and flavonoid content. In general, however, knowledge about the influence of seasonal variations in the accumulation of various metabolites in *T*. *hemsleyanum* tubers is very limited.

In this study, *T*. *hemsleyanum* tubers were collected semi-monthly and the key meteorological data were recorded for a year. Several individual flavonoids content in the tubers were sequentially analyzed and their biosynthetic pathways were interpreted. Furthermore, the contents of three major metabolites were determined and evaluated their ability to scavenge DPPH radical was evaluated. Correlations among the meteorological parameters, metabolite content, and their antioxidant capacities with seasonal variation were verified. Overall, this study not only provides an insight into the biosynthetic mechanism of flavonoids, but also helps understand the qualitative and quantitative variations in different constituents with environmental factors. This would assist in establishing scientific guidelines to regulate environmental factors influencing the production of desired metabolites in *T*. *hemsleyanum*, and help determine the geographical and climatic zones suitable for the large-scale artificial cultivation of this endangered plant.

## 2. Methods

### 2.1 Sampling of plant materials and acquisition of meteorological data

*T*. *hemsleyanum* is a member of the grape family Vitaceae with thin stems, leaves alternate and three-leaved compound leaves. And the root tubers are spindle shaped or elliptical. *T*. *hemsleanum* naturally grows in the wild and generally starts forming sizable tubers in the late stages of the second growth year. Morphological features of the *T*. *hemsleyanum* were identified by Prof. Zhang Jianhong, Institute of Forestry, Ningbo Academy of Agricultural Science, China. According to the randomized block trial design, the tubers of *T*. *hemsleanum* with identical growth age were collected semi-monthly from September 1 to August 31 for three consecutive years: 2016–2017, 2017–2018, and 2018–2019. The *T*. *hemsleyanum* tubers collected annually from September 1, 2017, to August 31, 2018, were provided by Sheng-wang Biotechnology Co., Ltd. Ningbo, China, and were incorporated in this work. After drying in an electro-thermostatic blast oven (Fuma Test Equipment Co., Ltd, China) at 60°C, the tuberous materials were finely powdered and stored in a sealed brown container at 4°C for further use.

The PhyTalk system (PhyTech Ltd., Israel) was used to automatically collect meteorological parameters during sampling [17]. Semi-monthly average data of meteorological factors, including average precipitation (P_ave_), mean temperature (T_m_), average maximum temperature (T_max_), minimum temperature (T_min_), average relative humidity (H_rel_), average minimum humidity (H_min_), and average sunshine duration (S_dur_) were simultaneously measured and stored.

### 2.2 Chemical reagents and solution

Isoorientin (luteolin-6-C-glucoside, IsoO), orientin (luteolin-8-C-glucoside, Or), rutin (Quercetin-3-o-rhamnosylglucoside, Rut), isoquercitrin (Quercetin-3-o-glucoside, IsoQ), kaempferol-3-0-rutinoside (Km3rut), quercetin (Qu), and apigenin (Ap) were purchased from the National Institute for the Control of Pharmaceutical and Biological Products, Beijing, China. Astragalin (kaempferol-3-0- glucoside, Ast) and kaempferol (Km) were obtained from Yuanye Biological Technology Co., Ltd. (Shanghai, China). Analytical grade anhydrous diethyl ether, ethyl acetate, petroleum ether, methanol, ethanol, aluminum nitrate, sodium hydroxide, sodium nitrite, formic acid, concentrated sulfuric acid, iron (III) chloride hexahydrate, glucose, and β-sitosterol (analytical grade) were purchased from Sinopharm Chemical Reagent Co., Ltd. Shanghai, China. HPLC-grade acetonitrile and methanol were purchased from Tedia Company, Inc. USA.

Individual flavonoids were dissolved in different amounts of acetone (HPLC grade) to generate a set of standard stock solutions: 2.0, 0.8, 3.0, 2.8, 3.1, 3.5, 0.8, 1.3, and 1.8 mg/mL, and each solution was progressively diluted into eight different concentration gradients. All solutions were stored at 4°C prior to UPLC analysis.

### 2.3 UPLC analysis of individual flavonoids

Dry powder of *T*. *hemsleyanum* tuber (5 g) was extracted by refluxing with 75 mL of 80% methanol at 85°C for 120 min. The supernatants from the two repeated extractions were mixed and evaporated in a rotary evaporator at 65°C. Thereafter, the as-obtained extract was dissolved in a binary solvent (55 mL) containing water/methanol at 10: 1 (mL/mL) and mixed with triple volumes (165 mL) of anhydrous diethyl ether and ethyl acetate, respectively. The extraction procedure was repeated twice, and the supernatants of anhydrous diethyl ether extraction and ethyl acetate extraction were combined, and the solvents in the mixture were removed by rotary evaporation to obtain flavonoids. Finally, the product was dissolved in 10 mL methanol and filtered through a 0.22 μm nylon filter for UPLC analysis.

*T*. *hemsleyanum* tuber extract was quantitatively analyzed using an ultrahigh-performance liquid chromatography system (UPLC) (Agilent 1290, Santa Clara, CA, USA). Chromatographic conditions included an injection volume of 10 μL, flow rate of 1 mL/min, column temperature of 32°C, detection wavelength of 280 nm, and column of 1.8 μm, 4.6 mm ×100 mm, i.d. (Agilent Technologies, Beijing, China), with gradient elution using 0.1% formic acid (A) and acetonitrile (B) as the mobile phase. By comparing the corresponding reference compounds, a total of nine individual flavonoids were identified and quantified with regression equations: IsoO, *y* = 10.06*x−*132.7 or *y* = 6.55*x−*5.63; Rut, *y* = 3.25*x−*9.24; IsoQ, *y* = 0.09*x−*36.2; Km3rut, *y* = 6.17*x*+ 2.0; Qu, *y* = 2.71*x*+11.9; Ap, *y* = 16.19*x−*231.45; Ast, *y* = 9.11*x*+12.4; and Km, *y* = 4.25*x*+15.0, (all *r*^*2*^ = 0.99), where *y* is the chromatographic peak area and *x* is the concentration (μg/mL).

### 2.4 Extraction and quantification of metabolites in *T*. *hemsleyanum*

#### 2.4.1 Measurement of phenolic compounds

The extract solution of *T*. *hemsleyanum* tuber was prepared according to the procedures described in Section 1.3. After the solution was concentrated by vacuum evaporation, the phenolic content was determined using the method described by Milbury [32] with minor modifications. First, 0.3 mL of the concentrated extract was added to a test tube containing 2.7 mL ethanol, following which 1 mL of 5% sodium nitrite solution was added. After standing for 6 min, the mixture was combined with 1 mL of 10% aluminum nitrate solution. After standing for another 6 min, the combined mixture was treated with 10 mL of 4% sodium hydroxide solution, followed by 10 mL of distilled water. After 15 min, the absorbance of the final mixture was measured at 510 nm using a UV-1800 spectrophotometer (Shimadzu, Japan). A standard curve regression of Rut (*y* = 0.0121*x*-0.0098, *r^2^* = 0.99) was used to estimate the sample concentration, and the content of phenolic compounds was expressed as Rut equivalents (mg/g dry weight). All samples were tested in five replicates to obtain data with *p*<0.05.

#### 2.4.2 Measurement of polysaccharides

An aqueous solution (50 mL) containing 2 g *T*. *hemsleyanum* tuber powder was mixed with a certain amount of glacial acetic acid to obtain a pH of 3, and the mixture was then extracted by refluxing at 82°C for 1.5 h. Next, the extraction solution was centrifuged at 3500 rpm for 15 min, and the supernatant (0.2 mL) was used to determine the polysaccharide content using a UV-1800 spectrophotometer (Shimadzu, Japan) at 490 nm using the phenol-sulfuric acid method [33]. Finally, the determined amount was compared with a standard regression curve of glucose (*y* = 0.0629*x*+0.0047, *r^2^* = 0.9995). All samples were measured in five repetitions to obtain data with *p*<0.05.

#### 2.4.3 Measurement of steroids

Steroids in the dry powder of *T*. *hemsleyanum* tuber were extracted with ethanol as a solvent by ultrasonication, followed by centrifugation at 2500 rpm for 8 min. Then, 20 mL of the sample solution was mixed with triple volume of petroleum ether (60 mL), and the supernatant was analyzed by the sulfate-phosphate-ferric method (SPF) [34] with minor modifications. Briefly, the chromogenic reagent of SPF was first prepared by dissolving 2.5 g FeCl_3_ 6H_2_O in 100 mL of 85% phosphoric acid, of which 4 mL solution was redissolved in 100 mL sulfuric acid for further use. Subsequently, 6 mL assay mixture containing 0.2 mL of ethanol extracted phytosterols and 3 mL SPF chromogenic solution was homogenized by shaking, and then analyzed at 560 nm using a UV-1800 spectrophotometer (Shimadzu, Japan). Finally, the steroid content was determined by comparing with a standard regression curve of beta-sitosterol (*y* = 0.0125*x*+0.0256, *r*^*2*^ = 0.99). All samples were analyzed in five repetitions to obtain data with *p*<0.05.

### 2.5 Determination of the activity to scavenge DPPH radical

The antioxidant activity of a compound or metabolite can be evaluated by various methods such as DPPH (2, 2-diphenyl-1-picrylhydrazyl)-RSA, ABTS-RSA, FRAP, and CUPRAC (cupric reducing antioxidant capacity) assay. To conduct a highly sensitive and rapid analysis, both DPPH and FRAP assays were preliminarily selected to evaluate the antioxidant activity of major metabolites in *T*. *hemsleyanum*, while DPPH assay showed better consistency and repeatability of evaluation. The capacity of a substance to scavenge DPPH radicals was determined using the spectrophotometric method described by Mohapatra *et al*. [35] with minor modifications.

All tests were conducted on a 96-well plate (400 μL). First, three concentrated extracts of phenolics, polysaccharides, and steroids were prepared from *T*. *hemsleyanum* tuber powder. For each of the three extracts, a series of samples with the same concentrations were prepared in methanol, and 0.1 mL each sample (25 μg/mL) was mixed with 3.9 mL of fresh solution of DPPH and methanol. L-ascorbic acid and methanol were used as positive and negative controls, respectively. After incubating at room temperature in dark for 30 min, the absorbance of the mixture was measured at 516 nm. The DPPH radical scavenging activity was repeated five times (*p*<0.05). Before the measurements, the standard regression equation for DPPH (*y* = 0.0317*x−*0.0022, *r*^2^ = 0.99) was determined using UV-1800. The percentage of DPPH inhibition was calculated using the following equation:

$$Percentage\;of\;DPPH\;inhibition=\frac{A_{blank}-A_{sample}}{A_{blank}}\times 100\%$$ (1)

*A*_*blank*_ = absorbance of blank at t = 0, *A*_*sample*_ = absorbance of the sample at t = 30 min.

### 2.6 Data processing and statistical analysis

Data on various environmental factors, the contents, and antioxidant activities of phenolic compounds, polysaccharides, and sterols in *T*. *hemsleyanum* tuber are expressed as mean ± standard deviation. After verifying the normality of the data, the insignificant differences were examined by both single and multi-factor ANOVA with SPSS software (version 20.0; IBM, Armonk, NY, USA). In addition, redundancy analysis of constrained ordination was performed using the software Canoco 5.0 (Microcomputer Power, New York, USA) to reveal the correlations among environmental factors, metabolites, and antioxidant activities. Moreover, Pearson correlation and Pearson partial correlation analyses were performed. The significance level of variation is assessed by the value of p, i.e., *p*<0.05 and *p*<0.01 indicate significant and very significant relations, respectively. The direction of correlation is determined by the symbol of coefficient, i.e., the symbol “+” and “−,” which represent positive correlation and negative correlation, respectively. The strength of correlation depends on the absolute value of correlation coefficient, i.e., the larger the coefficient, the stronger the correlation, and *vice versa*.

## 3. Results

### 3.1 Semi-monthly variation of meteorological factors

**Fig 1:**
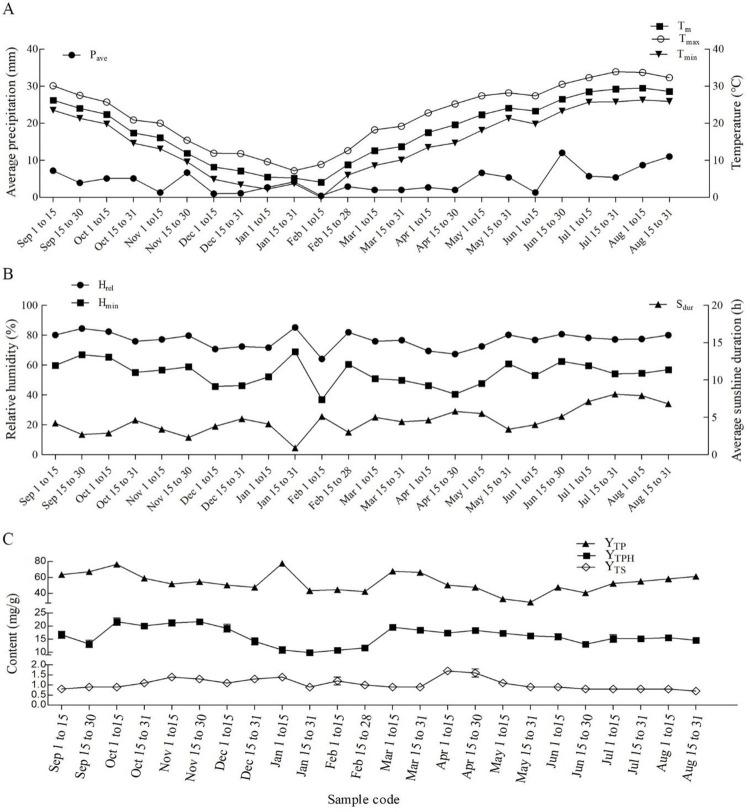
Contents of secondary metabolites in *T*. *hemsleyanum*and data of climatic factors. (A) Semi-monthly variation of precipitation and temperature; (B) Semi-monthly variation of humidity and sunshine duration; (C) Semi-monthly accumulation of three major metabolites. P_ave_, average precipitation; T_m_, mean temperature; T_max_, average maximum temperature; T_min_, mean minimum temperature; H_rel_, average relative humidity; H_min_, average minimum relative humidity; S_dur_, average sunshine duration; Y_TPH_, content of phenolic compounds, Y_TS_, content of sterols, Y_TP_, content of polysaccharides.

The average meteorological factor data were collected semi-monthly in the second growing year of *T*. *hemsleyanum* from, from September 1, 2017 to August 31, 2018. In total, 168 data including P_ave_, T_m_, T_max_, T_min_, H_rel_, H_min_, and S_dur_ are presented in Fig 1A and 1B. Each of these factors fluctuated seasonally within a certain range. P_ave_ reached a maximum of 12.0 mm in late July and a minimum of 0.4 mm during early February, T_m_ or T_min_ reached a maximum of 29.5 or 26.3°C in early August and a minimum of 4.1 or 0°C during early February, respectively, and T_max_ reached a maximum and minimum of 33.9 and 7.2°C in late July and late January, respectively; H_rel_ or H_min_ reached a maximum of 85.2 or 68.9% in late January and a minimum of 64.1 or 36.9% in early February; and S_dur_ reached a maximum of 8.1 h in late July and a minimum of 0.9 h in late January. In general, the summer from June to August was hot, rainy, and full of sunshine, whereas the winter from January to February was cold, with less rain and no sunshine; air humidity was the highest in the second half of January and the lowest in the first half of February.

### 3.2 Influence of seasonal climate on biosynthesis of flavonoids

**Fig 2:**
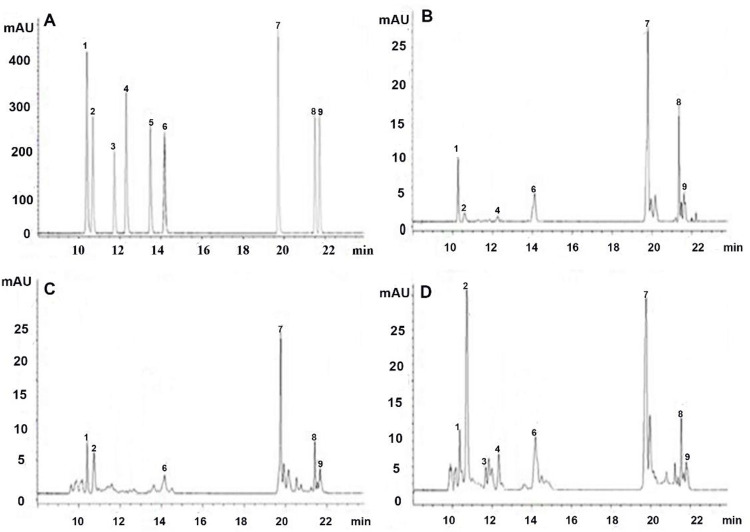
UPLC (280 nm) chromatograms of various samples: (A) standard sample with peak identification of components (1) IsoO(10.4 min), (2) Or (10.7 min), (3) Rut (11.7 min), (4)IsoQ (12.3 min), (5) Km3 (13.5 min), (6) Ast(14.2 min), (7) Qu(19.7 min), (8) Ap(21.5 min) and (9) Km(21.7 min); (B) sample on October 15, 2017; (C) sample on January 15, 2018; (D) sample on April 30, 2018.

Nine important individual flavonoids, including IsoO, Or, Rut, IsoQ, Km3rut, Ast, Qu, Ap, and Km were identified, and the UPLC chromatogram of the standard sample is shown in Fig 2A. Overall, the contents of nine flavonoid compounds in the 24 samples of *T*. *hemsleyanum* tubers collected semimonthly were determined. The UPLC chromatogram of three samples collected on October 15, 2017, January 15, 2018, and April 30, 2018 are presented in Fig 2B–2D, respectively.

#### 3.2.1 Seasonal dynamic accumulation of total flavonoids

The total content of the nine flavonoid compounds in *T*. *hemsleyanum* was evaluated semi-monthly. As displayed in Table 1, the total content considerably varied within a year, reaching a maximum of 394.5 μg/g in the second half of April and declining to a minimum in the first half of August. Based on statistical evaluation of the measured contents, the 24 semi-monthly samples of *T*. *hemsleyanum* could be divided into three groups: the first group with high content (a~c) that includes 4 samples from April 1 to May 31; the second group with medium content (d–h) that includes seven samples from December 16 to March 31 and two samples in June; and the third group with low content (i–p) that includes 11 samples from July 1 to December 15. The total flavonoid content in the first sample group fluctuated between 282 and 394.5 μg/g, and varied in the range 163–229 μg/g in the second sample group, and fluctuated below 122 μg/g in the third sample group. Such seasonal variation in flavonoid content can be attributed to the synergistic effects of various environmental factors (Fig 1). The combining conditions of P_ave_ (2.0~6.6 mm), T_m_ (17.5~24.1°C), H_rel_ (67.3~80.2%), and S_dur_ (3.4~5.8 h) favored the accumulation of flavonoids in *T*. *hemsleyanum*, whereas high P_ave_ (8.7 mm), T_m_ (29.5°C), H_rel_ (77.4%), and long S_dur_ (7.9 h) severely suppressed the accumulation. The seasonal variation of the total content resulted synthetically from changes in individual flavonoids, and therefore the seasonal variation affecting each individual flavonoid compound in *T*. *hemsleyanum* was further examined.

**Table 1:** Total content and individual content of nine flavonoids identified in *T*. *hemsleyanum* from 1 September 2017 to 31 August 2018.

Sample Code	Path Ⅰ (μg/g)			Path Ⅱa(μg/g)				Path Ⅱb(μg/g)		Total sum (μg/g)
	IsoO	Or	Ap	Rut	IsoQ	Qu	Km	Km3rut	Ast	
1to 15Sep2017	--	5.5±0.1^qr^	15.4±1.6^cde^	--	--	12.6±0.8^o^	--	5.0±0.4^b^	6.5±0.4^n^	44.6
15 to 30Sep 2017	--	4.6±0.1^r^	15.6±1.4^bcd^	--	--	17.9±0.7^m^	--	6.2±0.5^a^	9.2±0.6^m^	52.8
1 to 15Oct 2017	14.4±0.6^h^	36.1±1.2^g^	15.8±1.5^bc^	--	--	24.1±1.3^l^	3.7±0.3^f^	--	27.9±1.7^e^	121.3
15 to 31Oct 2017	--	16.0±0.6^n^	15.4±1.0^cde^	7.6±0.4^i^	--	--	--	--	2.7±0.2^r^	41
1 to 15Nov 2017	--	22.1±0.5^l^	15.4±0.8^cde^	15.7±0.9^d^	--	12.5±0.2^o^	--	--	--	64.8
15 to 30Nov 2017	14.7±0.5^gh^	23.2±0.8^k^	15.9±0.8^b^	--	--	37.0±3.5^i^	2.8±0.2^g^	--	11.7±0.7^k^	103.5
1 to 15Dec 2017	15.5±0.7^fg^	26.0±0.8^j^	15.4±0.9^cde^	12.9±1.2^e^	--	15.2±1.3^n^	--	--	1.5±0.1^t^	84.5
15 to 31Dec 2017	86.7±3.2^b^	78.7±3.0^a^	--	12.9±1.2^e^	12.4±1.2^ij^	15.4±1.2^n^	--	--	11.1±0.9^l^	216.1
1 to 15Jan 2018	16.1±0.4^f^	28.9±0.6^i^	--	12.1±1.1^f^	--	120.5±5.8^e^	7.3±1.3^d^	--	0.9±0.02^u^	184.6
15 to 31Jan 2018	19.2±0.8^e^	33.4±1.0^h^	--	--	--	140.6±6.4^c^	9.2±1.6^b^	--	3.4±0.2^q^	204.9
1 to 15Feb 2018	14.8±0.5^gh^	33.5±1.1^h^	--	21.3±1.7^b^	16.9±1.3^g^	27.9±1.3^j^	--	--	71.8±4.5^a^	185.5
15 to 28Feb 2018	14.7±0.3^gh^	42.4±1.0^e^	--	21.7±1.8^b^	25.0±1.4^c^	26.5±1.4^k^	2.4±0.1^g^	--	31.3±2.7^c^	162.1
1 to 15Mar 2018	16.2±0.3^f^	39.6±0.9^f^	--	--	12.2±1.2^ij^	87.5±5.7^g^	4.8±0.3^e^	--	2.4±0.08^rs^	161.5
15 to 31Mar 2018	16.1±0.3^f^	79.2±2.4^a^	--	8.7±0.6^h^	18.6±1.3^e^	57.6±3.9^h^	--	--	5.4±0.4^o^	184.9
1 to 15Apr 2018	24.0±0.4^c^	59.5±3.0^b^	18.2±0.7^a^	9.4±0.6^g^	17.7±1.3^f^	144.4±7.6^b^	8.5±0.5^c^	--	14.5±0.8^i^	292.7
15 to 30Apr 2018	119.6±4.5^a^	25.3±0.7^j^	18.1±0.8^a^	26.4±2.5^a^	28.8±1.5^b^	150.8±8.9^a^	10.3±1.3^a^	--	15.2±1.4^h^	392.8
1 to 15May 2018	19.3±0.5^e^	51.4±1.4^c^	14.9±0.9^f^	19.8±1.8^c^	30.6±1.5^a^	124.9±6.4^d^	--	--	21.3±1.9^f^	281.3
15 to 31May 2018	21.1±0.5^d^	50.2±1.0^d^	15.0±0.6^ef^	21.4±1.2^b^	20.9±1.4^d^	150.7±5.8^a^	2.7±0.06^g^	--	16.3±0.9^g^	297.1
1 to 15Jun 2018	16.2±0.3^f^	18.4±0.8^m^	--	13.3±1.1^e^	14.1±1.1^h^	111.4±5.8^f^	0.7±0.04^i^	--	54.6±3.2^b^	227.2
15 to 30Jun 2018	15.7±0.2^f^	23.0±1.4^kl^	--	8.6±0.7^h^	12.7±0.8^i^	111.4±6.9^f^	1.2±0.07^h^	--	29.3±1.4^d^	200.6
1 to 15Jul 2018	--	11.6±0.5^o^	15.9±0.6^b^	8.7±0.5^h^	11.7±0.6^j^	26.7±2.8^k^	--	--	13.0±0.7^j^	87
15 to 31Jul 2018	--	7.4±0.1^p^	15.3±0.5^def^	8.7±0.6^h^	10.8±0.4^k^	23.1±1.9^l^	--	--	5.9±0.3^o^	70.5
1 to 15Aug 2018	--	5.8±0.2^q^	--	7.8±0.4^i^	9.1±0.5^l^	7.3±0.4^q^	--	1.2±0.2^d^	2.0±0.1^st^	32.4
15 to 31Aug 2018	14.0±0.4^h^	7.6±0.1^p^	--	4.7±0.3^j^	8.3±0.6^m^	11.0±0.9^p^	--	2.9±0.2^c^	4.7±0.4^p^	51.8

Note: IsoO: isoorientin, Or: orientin, Rut: rutin, IsoQ: isoquerctin, Km3rut: kaempferol-3-0-rutinoside, Ast: astragalin, Qu: quercetin, Ap: apigenin, Km: kaempferol. The lower letter indicates significant difference in group (P<0.05).--: Data not detected.

#### 3.2.2 Seasonal dynamic content of individual flavonoids

The variation in the content of nine flavonoids from the 24 *T*. *hemsleyanum* samples are presented in Table 1. Individually, IsoO was identified in 21 samples, and showed a peak of 86.7μg/g during the second half of December and reached a maximum of 119.6 μg/g in the second half of April; Or was present in all 24 samples, showing a peak of 78.7μg/g in the second half of December and reached the maximum of 79.2 μg/g in the second half of March; Rut at <26.5 μg/g was present in 18 samples; IsoQ at < 30.7μg/g was observed in 15 samples; Km3rut at concentration < 6.5 μg/g was found only in 4 samples collected during August and September; Ast was identified in 23 samples, showing a peak of 54.6 μg/g in the first half of June and reached a maximum of 71.8 μg/g in the first half of February; Qu was available in 23 samples, with a concentration of over 111 μg/g in January, April, May, and June, and reached a maximum of 150.8 μg/g in the second half of April; Ap at concentrations between 14.9 and 18.2 μg/g was found in 13 samples; and Km at <10.5 μg/g was found in 11 samples. Among these individual flavonoids, Qu was the highest in most months, and especially accumulated at high levels in January, April, May, and June, while Km3rut was hardly generated or accumulated during most months.

From the data in Table 1, the total content of flavonoids reached higher levels between 281.3 and 392.8 μg/g from April to May, and the contributions of nine individual flavonoid compounds were significantly different and seasonally varied. For example, IsoO, Ast, and Qu at 7.9, 38.6, and 15.0% contributed to the highest total accumulation of flavonoids (392.8 μg/g) in the second half of April, at 7.9, 38.6, and 15.0% contributed to the total accumulation (186.2 μg/g) in the first half of February, and at 0, 6.0, and 22.0% contributed to the lowest total accumulation (33.2 μg/g) in the first half of August, respectively. Such dynamic distributions of various flavonoid compounds during different seasons provides an insight into their biosynthetic mechanisms in *T*. *hemsleyanum* and diversity in their response to the environment.

### 3.3 Seasonal dynamics of accumulation and activity to scavenge DPPH radical of metabolites

#### 3.3.1 Contents of three major metabolites

The estimation of phenolic compounds (Y_TPH_), sterols (Y_TS_), and polysaccharides (Y_TP_) in 24 samples of *T*. *hemsleyanum* tubers provided 72 data in total. As shown in Fig 1C, their contents in any sample followed the order of Y_TPH_> Y_TP_> Y_TS_, and varied in the range of 29.1±1.2~77.7±3.2 mg/g, 9.9±0.9~21.7±0.9 mg/g, and 0.73±0.09~1.7±0.1 mg/g, respectively. Polysaccharides were the most abundant, and sterols were rarely found in *T*. *hemsleyanum*. Y_TPH_ content was notably contributed to by various phenolic compounds, including flavonoids and phenolic acids, and thus its seasonal variation differed from that in the total content of individual flavonoids (Table 1).

#### 3.3.2 DPPH radical scavenging activity of three major metabolites

Table 2 presents the data of 72 DPPH radical scavenging activity for three major metabolites in 24 samples of *T*. *hemsleyanum* tubers. Throughout the year, the DPPH radical scavenging rate (%) of phenolic compounds (Z_TPH_), sterols (Z_TS_), and polysaccharides (Z_TP_) varied in the range of 39.9–95.1%, 35.4–43.5%, and 5.5–9.8%, respectively, but constantly followed the order of Z_TPH_> Z_TP_> Z_TS._ Sterols with minimal content clearly displayed considerably lower antioxidant activity than those of phenolic compounds or polysaccharides.

**Table 2:** DPPH radical scavenging rates of phenolic compounds, sterols and polysaccharides.

	The DPPH radical scavenging				The DPPH radical scavenging		
Sample Code	Z_TPH_ (%)	Z_TS_ (%)	Z_TP_ (%)	Sample Code	Z_TPH_ (%)	Z_TS_ (%)	Z_TP_ (%)
1to 15Sep2017	62.2±2.2^f^	5.8±0.2^g^	41.7±0.7^b^	1 to 15Mar 2018	64.1±1.1^f^	9.8±0.2^a^	39.4±1.2^c^
15 to 30Sep 2017	51.5±1.8^gh^	6.2±0.3^f^	42.2±1.1^ab^	15 to 31Mar 2018	82.2±2.1^b^	9.5±0.3^a^	38.9±2.2^c^
1 to 15Oct 2017	67.6±2.6^e^	6.5±0.3^e^	43.5±1.4^a^	1 to 15Apr 2018	80.4±2.9^b^	7.2±0.2^d^	36.9±2.6^e^
15 to 31Oct 2017	39.9±1.4^i^	7.3±0.5^d^	41.0±0.6^b^	15 to 30Apr 2018	95.1±2.3^a^	6.6±0.3^e^	35.4±1.3^f^
1 to 15Nov 2017	60.8±1.8^f^	8.5±0.4^b^	39.7±2.2^c^	1 to 15May 2018	80.0±1.2^b^	6.3±0.4^ef^	38.2±1.5^d^
15 to 30Nov 2017	51.6±2.4^gh^	8.4±0.2^b^	39.2±1.7^c^	15 to 31May 2018	93.2±0.9^a^	5.9±0.2^f^	38.0±1.8^d^
1 to 15Dec 2017	71.9±2.6^d^	7.6±0.3^c^	39.4±1.4^c^	1 to 15Jun 2018	64.3±1.9^f^	6.0±0.3^f^	39.0±0.8^c^
15 to 31Dec 2017	76.9±2.2^c^	8.2±0.1^c^	38.9±1.8^d^	15 to 30Jun 2018	55.8±2.7^g^	5.7±0.3^g^	39.1±0.4^c^
1 to 15Jan 2018	52.6±2.5^g^	8.9±0.3^b^	40.7±1.3^bc^	1 to 15Jul 2018	47.4±2.2^h^	5.8±0.1^g^	39.8±0.6^c^
15 to 31Jan 2018	64.0±1.7^f^	6.6±0.1^e^	37.9±0.8^d^	15 to 31Jul 2018	54.4±1.0^g^	5.5±0.2^h^	39.3±1.1^c^
1 to 15Feb 2018	49.5±1.6^h^	7.7±0.4^c^	42.3±1.3^ab^	1 to 15Aug 2018	61.1±2.3^f^	9.8±0.1^a^	40.8±1.3^b^
15 to 28Feb 2018	68.7±2.2^e^	7.0±0.2^d^	40.1±1.7^c^	15 to 31Aug 2018	77.9±1.5^c^	9.5±0.1^a^	41.3±0.6^b^

*Note*: Z_TPH_ (DPPH radical scavenging rates of phenolic compounds), Z_TS_ (DPPH radical scavenging rates of sterols), Z_TP_ (DPPH radical scavenging rates of polysaccharides). Different letters indicate that there is a significant difference between the data at p <0.05.

## 4. Discussion

### 4.1 Biosynthesis of various flavonoids in *T*. *hemsleyanum*

Secondary metabolism in plants generates flavonoids, and more than 9000 flavonoid compounds have been discovered to date [36]. Flavonoids possess diverse bioactivities and potential medicinal values, such as antimicrobial, anticancer, antibacterial and antiinflammation [37–39]. There is an academic interest in quantitatively interpreting the biosynthetic mechanism of the major individual flavonoid in *T*. *hemsleyanum*.

#### 4.1.1 Biosynthetic reaction network

**Fig 3:**
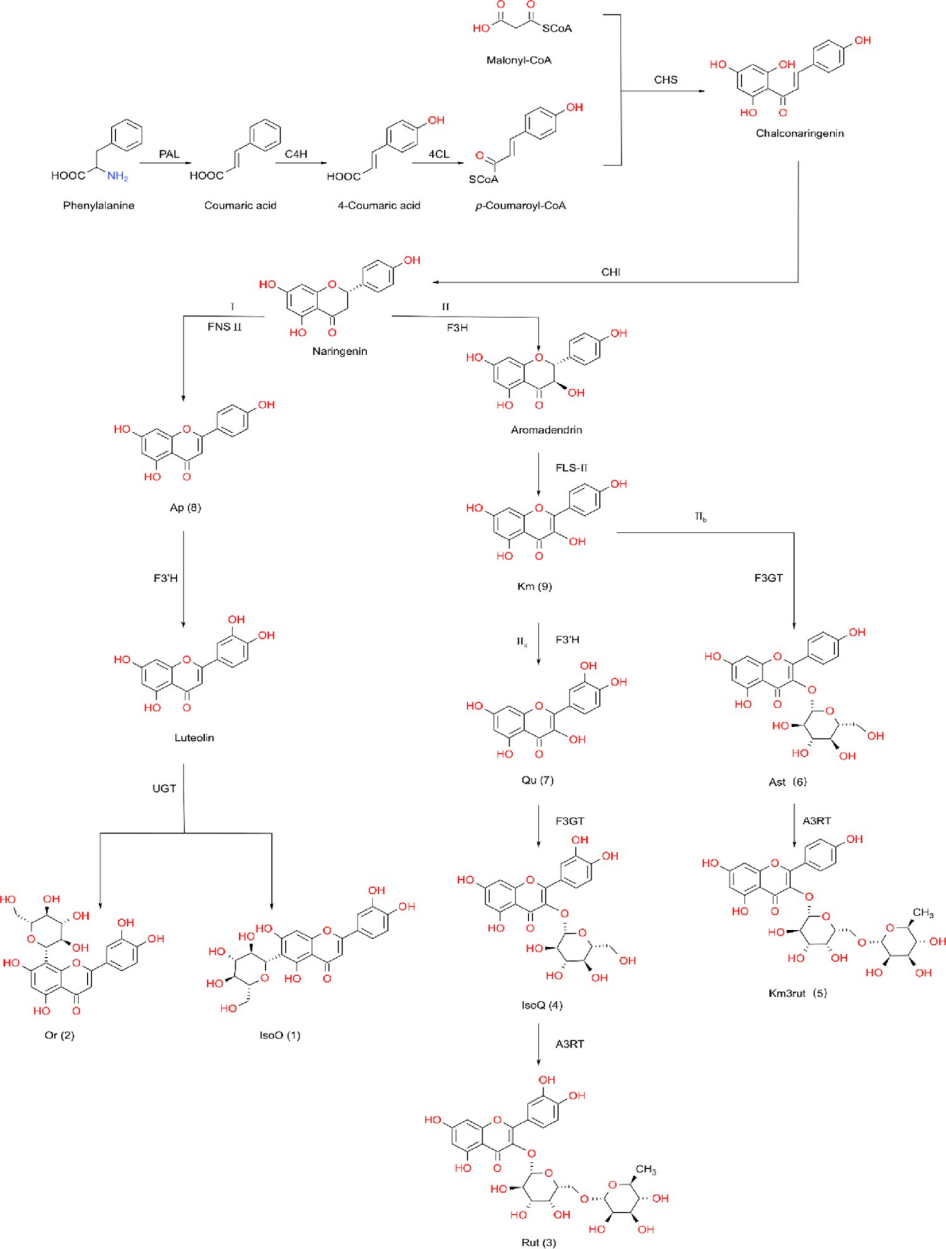
A schematic outline of flavonoid pathway proposed for *T*. *hemsleyanum*. PAL, phenylalanine ammonia-lyase; C4H, trans-cinnamate 4-monooxygenase; 4CL, 4-coumarate-CoA ligase; CHS, chalcone synthase; CHI, chalcone isomerase; FNS II, flavone synthase II; F3′H, flavonoid 3′-hydroxylase; F3H, flavonone 3-hydroxylase; FLS-Ⅱ, flavone synthase Ⅱ; UGT, UDP-glucosyltransferase; F3GT, flavonol-3-O-glucosyltransferase; A3RT, anthocyanidin-3-O-glucoside-6-O-rhamnosyltransferase.

Considering their core structural unit, various flavonoids can be derived from three basic compounds: flavanone, flavone, and flavonol. According to the core structures of nine individual flavonoids identified in *T*. *hemsleyanum* (Fig 2 or Table 1) and referring to the flavonoids biosynthesized in other plants [40–43], the biosynthetic route network of flavonoids in *T*. *hemsleyanum* is shown in Fig 3. Focusing on 4’5,7-trihydroxyflavanone (naringenin*)* as the central intermediate to bridge the upstream and downstream, the entire reaction chain may be roughly divided into two stages: preceding and proceeding. In the preceding stage, the enzymes, including phenylalanine ammonialyase (PAL), trans-cinnamate 4-monooxygenase (C4H), and 4-coumarate-CoA ligase (4CL), sequentially catalyze a series of conversion reactions from phenyalanine to*p*-coumaroyl-CoA, and chalcone synthase (CHS) catalyzes the reaction between *p*-coumaroyl-CoA and malonyl-CoA to generate naringenin chalcone, which undergoes stereospecific cyclization over chalcone isomerase (CHI) to form naringenin. In the proceeding stage, the as-formed naringenin is first dehydrogenated by flavone synthase II (FNS II) to form 4’,5,7-trihydroxyflavone (Ap) or hydroxylated by flavonone 3-hydroxylase(F3H) to form 3,4’,5,7-tetrahydroxy flavanone (aromadendrin, Aro). Subsequently, two parallel routes of downstream reactions are initiated: the path I generates flavone derivatives and path II generates flavonol derivatives. Along path I, the as-formed Ap could be hydroxylated by flavonoid 3-hydroxylase (F3’H) to generate 3’,4’5,7-tetrahydroxyflavone (luteolin, Lu), which is rapidly glycosylated by UDP-glucosyltransferase (UGT) to form a pair of competitive isomers: Or and IsoO. Along Path II, the as-formed aromadendrin is first dehydrogenated by flavone synthase II (FLS-II) into 4’,5,7-trihydroxyflavonol (Km) and then branched into two parallel sub-routes, including path IIa that produces Qu, IsoQ, and Rut in series and path IIb that produces Ast and Km3rutin.

The above biosynthetic network clearly revealed the elementary reactions originating from naringenin in the synthesis of four terminal flavonoids (Or, IsoO, Rut, and Km3rut), and the formation and conversion of seven intermediate flavonoids (Ap, Lu, Aro, Km, Qu, IsoQ, and Ast) in *T*. *hemsleyanum*. The formation or conversion of each individual flavonoid involves its own elementary reaction catalyzed by a specific enzyme, and different flavonoids or enzymes would exhibit different reactivities or catalytic activities. The rate and selectivity of every elementary reaction would kinetically depend on both the reactivity of the reactant flavonoid and the activity of the participating enzyme. Among all the intermediates, Lu in path I and Aro in path II remained undetected in *T*. *hemsleyanum* (Table 1), probably because of their considerably high reactivity, causing rapid conversion into downstream products.

#### 4.1.2 Biosynthetic path selectivity

To quantitatively interpret and compare the biosynthetic reactions of various flavonoids in *T*. *hemsleyanum*, the production selectivity of the jth flavonoid compound (PS_j_) was first defined as,

$${PS}_{j}=\frac{Y_{j}}{\sum_{j=1}^{9}Y_{j}}$$

where Y_j_ is the content of the jth flavonoid (j = 1, 2, …., 9) in Table 1.

Kinetically, PS_j_ can be described as the relative rate of the jth product formation after the overall reaction rate and is normalized, and thus, is adopted to compare the phenomenokinetic selectivity of various flavonoids. In the second half of April, for instance, the PS of IsoO, Or, Rut, IsoQ, Km3rut, Ast, Qu, Ap, and Km in *T*. *hemsleyanum* were calculated to yield values 0.30, 0.06, 0.07, 0.07, 0.00, 0.04, 0.38, 0.05, and 0.03, respectively.

From the perspective of the micro-reaction mechanism, the PS data can also be used to quantitatively evaluate the kinetic selectivity of every elementary reaction in the formation or conversion of various flavonoids (Fig 3). In particular, it comparatively interprets the reaction path selectivity (RPS) of two parallel routes initiated by an intermediate flavonoid and their seasonal variations. According to the biosynthetic mechanism shown in Fig 3, the dehydrogenation and hydroxylation of naringenin originates from two competitive reaction routes: path I and path II; thus, their RPS can be evaluated using the following equations:

$$\begin{array}{l} {RPS}_{I}={PS}_{8}+{PS}_{1}+{PS}_{2}\\ {RPS}_{II}={PS}_{9}+{PS}_{7}+{PS}_{4}+{PS}_{3}+{PS}_{6}+{PS}_{5} \end{array}$$

**Fig 4:**
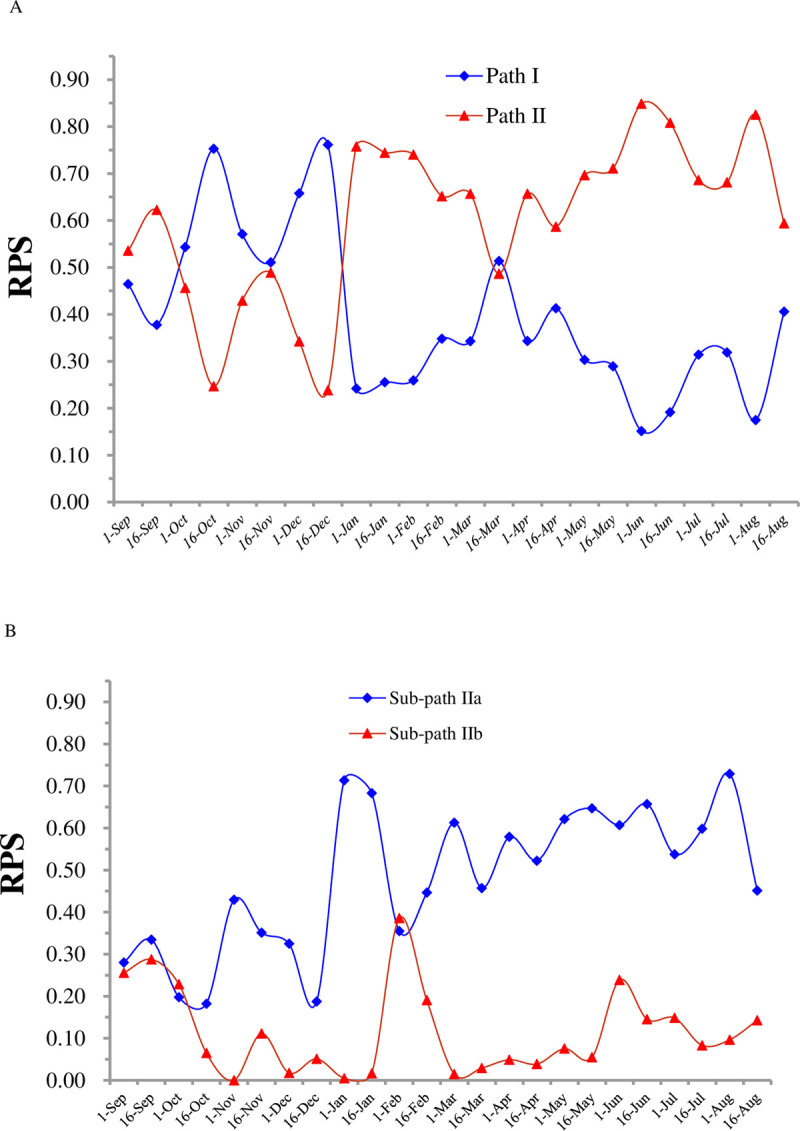
Reaction path selectivity (RPS) of flavonnoidbiosynthesis in *T*. *hemsleyanum* and their seasonal variations. (A) Path I: producing Ap, IsoO and Or; Path II: producing Km, Qu, IsoQ, Rut, Ast and Km3rut; (B) Sub-path IIa: producing Qu, IsoQ and Rut; Sub-path IIb: producing Ast and Km3rut.

The as-calculated data for RPS_I_ and RPS_II_ with seasonal variation are illustrated in Fig 4A. RPS_I_ was significantly higher than RPS_II_ during the period from October 1 to December 31, except for November 16–30, but RPS_II_ was considerably higher than RPS_I_ during the period of January 1 to September 30, except for March 16–31, while the value of RPS_I_ was substantially closer to that of RPS_II_ in the second half of November or second half of March. These results indicated that from January to September, except for mid-to-late March, naringenin considerably favored hydroxylation, thereby producing six flavonol compounds, but from October to December, except for mid-to-late November, naringenin possibly preferred dehydrogenation, thereby producing three flavone compounds.

As shown in Fig 3, the intermediate Km in path II could be hydroxylated by F3’H or glycosylated by F3GT to initiate the reaction sub-path IIa or IIb, respectively, and the selectivity of these two competitive reaction paths can be evaluated using the following formulas:

$$\begin{array}{l} {RPS}_{IIa}={PS}_{7}+{PS}_{4}+{PS}_{3}\\ {RPS}_{IIb}={PS}_{6}+{PS}_{5} \end{array}$$

The as-calculated data of RPS_IIa_ and RPS_IIb_ with seasonal variation are displayed in Fig 4B. The value of RPS_IIa_ was significantly higher than that of RPS_IIb_ from November to August except for February 1–15, indicating that in the most months in a year, the preferred path by Km was hydroxylation rather than glycosylation.

In addition, Or and IsoO are a pair of isomeric compounds from the glycosylation of the active intermediate Lu (Fig 3). As two terminal products in reaction path I, their production selectivity PS_2_ and PS_1_could be easily calculated from the yield data in Table 1, which shows that in most months in a year, glycosylation preferentially formed Or rather than IsoO.

Overall, diversity in the elementary reaction pathway and varying enzyme catalytic activity is essential for the biosynthesis of various flavonoids in *T*. *hemsleyanum*, and the production selectivity of each flavonoid compound depends on its synthetic pathway, enzyme activity, and environmental factors.

### 4.2 Correlation between major metabolites and environmental factors

Environmental factors such as light intensity, humidity and temperature would influence plant growth and secondary metabolite production [44, 45]. And several studies have investigated the influence of seasonal changes on the production of plant metabolites [21, 44], particularly their effect on the accumulation of certain specific compounds and biological activity in plants [18, 46–48]. According to statistical analysis of the observed or measured data, the correlations among environmental factors, the contents, and antioxidant activities of major metabolites in *T*. *hemsleyanum* have been evaluated and discussed below.

#### 4.2.1 Inter-correlation between metabolite contents and environmental factors

**Fig 5:**
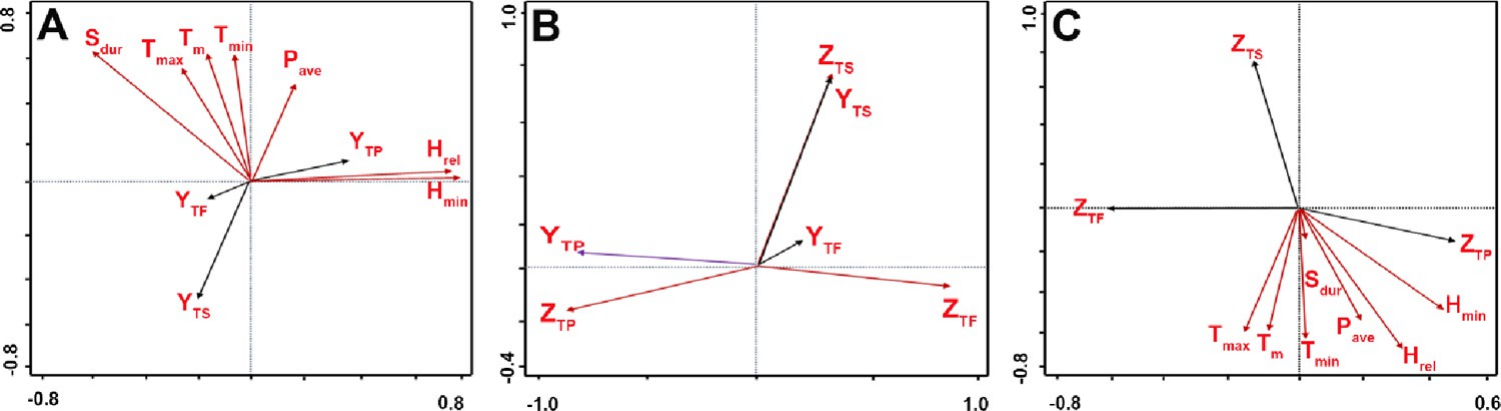
Redundancy analysis (RDA) plots on the contents of secondary metabolites, DPPH radical scavenging rates and environmental factors. (A) Contents of secondary metabolites and environmental factors; (B) DPPH radical scavenging rates and contents of secondary metabolites; (C) DPPH radical scavenging rates and climatic factors. P_ave_, T_m_, T_max_, T_min_, H_rel_, H_min_ and S_dur_indicated in Fig 1; Y_TPH_, Y_TS_ and Y_TP_ indicated in Fig 1; Z_TPH_, Z_TS_ and Z_TP_ indicated in Table 2.

Redundancy and Pearson analyses were performed to evaluate the correlation between the contents of three major metabolites (Y_TPH_, Y_TP_, and Y_TS_) and seven environmental factors (P_ave_, T_m_, T_max_, T_min_, H_rel_, H_min_, and S_dur_). The statistical significance of various variables and responses were ranked directionally, which enabled redundancy analysis to preliminarily evaluate the correlation or influence of various environmental factors toward or on metabolites. From the RDA plot in Fig 5A, most climatic factors were negatively correlated with the metabolites, and all correlation coefficients were less than 0.8. The data in Table 3 summarizes the results of Pearson correlation analysis and Pearson partial correlation analysis for the three response quantities (Y_TPH_, Y_TP_, Y_TS_) and the seven environmental variables. Y_TS_ was significantly correlated with more variable factors and with larger negative correlation coefficients than Y_TPH_ or Y_TP_, suggesting that the accumulation of sterols was significantly and negatively affected by solar illumination, temperature, humidity, and rainfall during the growth of *T*. *hemsleyanum*. Furthermore, Y_TS_ was considerably negatively and significantly correlated with each of environmental factors, but was positively correlated with T_m_ (*r* = 0.434, *p*<0.05) after deducting the effect of T_min_, indicating that higher humidity, higher and extremely low temperatures could inhibit sterol accumulation in *T*. *hemsleyanum*; therefore, controlled temperature (17.5~19.6°C) and soil moisture (40.5~46.2%) and shading could help accumulate sterols. Y_TPH_ was positively correlated with temperature (T_m_, T_max_, T_min_) or sunshine duration (S_dur_), but negatively correlated with precipitation (P_ave_) or humidity (H_rel_, H_min_), and therefore adjusting the temperature (15.4~25.7°C) and soil moisture content (55.1~65.4%) and illuminating at night could guarantee increased accumulation of phenolic compounds in *T*. *hemsleyanum*; additionally, Y_TP_ was positively correlated with each climatic factors, indicating that the controlled increase in temperature and soil moisture content could enhance the accumulation of polysaccharides in *T*. *hemsleyanum*.

**Table 3:** Pearson correlation analysis among DPPH radical scavenging rates and contents of metabolites and climatic factors.

Component	Factors	Correlation coefficients	Partial correlation coefficients of control variables						
			P_ave_	T_m_	T_max_	T_min_	H_rel_	H_min_	S_dur_
Y_TPH_	T_m_	0.189	0.306		0.157	0.222	0.232	0.242	–0.174
	T_max_	0.207	0.316	0.194		0.189	0.232	0.238	0.276
	T_min_	0.177	0.301	–0.142	–0.155		0.219	0.233	0.219
Y_TS_	P_ave_	–0.516^a^		–0.256	–0.300	–0.212	–0.309	–0.348	–0.483^b^
	T_m_	–0.534^a^	–0.299		–0.432^b^	0.434^b^	–0.431^b^	–0.452^b^	–0.507^a^
	T_max_	–0.499^b^	–0.261	0.381		0.390	–0.433^b^	–0.449^b^	–0.470^b^
	T_min_	–0.565^a^	–0.337	–0.476^b^	–0.482^b^		–0.429^b^	–0.458^b^	–0.540^a^
	H_rel_	–0.635^a^	–0.514^a^	–0.565^a^	–0.595^a^	–0.533^a^		–0.545^a^	–0.781^a^
	H_min_	–0.532^a^	–0.375	–0.449^b^	–0.488^b^	–0.409^b^	0.395		–0.697^a^
Y_TP_	P_ave_	0.095		0.186	0.205	0.169	–0.005	–0.045	0.131
	H_rel_	0.205	0.183	0.244	0.244	0.244		–0.230	0.187
	H_min_	0.273	0.261	0.312	0.309	0.314	0.292		0.261
Z_TPH_	--	--	–0.126	ns	0.069	ns	–0.188	–0.281	ns
Z_TS_	--	--	–0.54^b^	–0.567^a^	–0.532^a^	–0.597^a^	–0.634^a^	–0.532^a^	–0.247^b^
Z_TP_	--	--	0.186	ns	ns	ns	0.295	0.332	ns

*Note*: Y_TPH_ (content of phenolic compounds), Y_TS_ (content of sterols), Y_TP_ (content of (polysaccharides); Z_TPH_(DPPH radical scavenging rates ofphenolic compounds), Z_TS_ (DPPH radical scavenging rates ofsterols), Z_TP_ (DPPH radical scavenging rates ofpolysaccharides); P_ave_ (average precipitation), T_m_ (mean temperature), T_max_ (average maximum temperature), T_min_(mean minimum temperature), H_rel_ (average relative humidity), H_min_ (average minimum relative humidity), S_dur_ (average sunshine duration); ^a^shows significance at the level as *p* = 0.01; ns means insignificant, ^b^shows significance at the level as *p* = 0.05. --: Data not detected. ns: no significant.

#### 4.2.2 Inter-correlation of DPPH radical scavenging activity and environmental factors

The relationship between the antioxidant activities of major metabolites in *T*. *hemsleyanum* and environmental variables was also evaluated by redundancy analysis and Pearson analysis. The RDA plot in Fig 5C ranked directionally indicated a negative correlation between the DPPH radical scavenging rate of sterols (Z_TS_) and each of the environmental factors, a positive correlation between the DPPH radical scavenging rate of polysaccharides (Z_TP_) and each of these variables, and a negative correlation between the DPPH radical scavenging rate of phenolic compounds (Z_TPH_) and T_m_ or T_max_, but a positive correlation between Z_TPH_ and P_ave_, T_min_, H_rel_, H_min_, and S_dur_. Moreover, the data from the Pearson correlation analysis in Table 3 indicate that Z_TS_ was negatively and significantly correlated with P_ave_ (*r* = -0.54), T_m_ (*r* = -0.57), T_max_ (*r* = -0.53), T_min_ (*r* = -0.60), H_rel_ (*r* = -0.63), H_min_ (*r* = -0.53), and S_dur_ (*r* = -0.25), and either Z_TF_ or Z_TP_ showed an insignificant or no correlation with various climatic variables. These results suggest that increasing precipitation, temperature, and humidity could significantly reduce the antioxidant activity of sterols, but changes in environmental conditions had negligible effects on the antioxidant activity of phenolic compounds or polysaccharides in *T*. *hemsleyanum*.

#### 4.2.3 Inter-correlation on metabolite contents and DPPH radical scavenging activity

Several studies have shown a strong and significant correlation between the phenolic compound content and DPPH antioxidant activity [49]. Therefore, there is an increased interest in analyzing the correlation between the DPPH scavenging ability and variations in the concentration of the active component. The RDA plot in Fig 5B shows a considerably strong and positive correlation between the content of sterols (Y_TS_) and its DPPH radical scavenging rate (Z_TS_) with a correlation coefficient very close to 1, a strong and positive correlation between the content of polysaccharides (Y_TP_) and its DPPH radical scavenging rate (Z_TP_), and a positive but relatively weak correlation between the DPPH radical scavenging rate (Z_TPH_) and the content of phenolic compounds (Y_TPH_). In addition, Pearson’s analysis confirmed a positive and significant correlation between the DPPH radical scavenging rate, Y_TS_ (*r* = 0.998, *p*<0.01), and Y_TP_ (*r* = 0.899, *p*<0.01), suggesting that the antioxidant activities of sterols and polysaccharides can be significantly enhanced by increasing their contents.

## 5. Conclusion

Naringenin highly favored hydroxylation, and subsequently produced six flavonol compounds from January to September, except for mid-to-late March, and preferred dehydrogenation from October to December and derived three flavone compounds, except for mid-to-late November. The value of RPS_IIa_ was significantly higher than that of RPS_IIb_ during the period from November to August except for February 1–15, indicating that in most months of the year, Km preferred the path of hydroxylation rather than glycosylation.

## Abbreviations

ABTS: 2,2’-Azinobis-(3-ethylbenzthiazoline-6-sulphonate)
Ap: Apigenin
Ast: Astragalin
CUPRAC: Cupric Reducing Antioxidant Capacity
DPPH: 2, 2-diphenyl-1-picrylhydrazyl
FRAP: Benzie Ferric Reducing Ability Plasma
HPLC: High Performance Liquid Chromatography
H_min_: Average Minimum Humidity
H_rel_: Average Relative Humidity
IsoO: Isoorientin
IsoQ: Isoquercitin
Km: Kaempferol
Km3rut: Kaempferol-3-0-rutinoside
Lu: Luteolin
Or: Orientin
P_ave_: Average Precipitation
PS: Production Selectivity
Qu: Quercetin
RPS: Reaction Path Selectivity
RSA: Radical Scavenging Activity
Rut: Rutin
S_dur_: Average Sunshine Duration
T_m_: Mean Temperature
T_max_: Average MaximumTemperature
T_min_: Minimum Temperature
TPH: Phenolic Compounds
TP: Polysaccharids
TS: Sterols
UPLC: Ultra Performance Liquid Chromatography
